# NO Promotes Seed Germination and Seedling Growth Under High Salt May Depend on EIN3 Protein in *Arabidopsis*

**DOI:** 10.3389/fpls.2015.01203

**Published:** 2016-01-07

**Authors:** Xilong Li, Yajie Pan, Bowen Chang, Yucheng Wang, Zhonghua Tang

**Affiliations:** ^1^State Key Laboratory of Tree Genetics and Breeding, Northeast Forestry UniversityHarbin, China; ^2^The Key Laboratory of Plant Ecology, Northeast Forestry UniversityHarbin, China

**Keywords:** *Arabidopsis*, seed germination, salt stress, nitric oxide, ethylene

## Abstract

The gas molecule nitric oxide (NO) can cooperate with ethylene to tightly modulate plant growth and stress responses. One of the mechanism of their crosstalk is that NO is able to activate ethylene biosynthesis, possibly through post-translational modification of key enzymes such as ACC synthase and oxidase by *S*-nitrosylation. In this paper, we focus on the crosstalk of NO with ethylene signaling transduction transcription factor EIN3 (Ethylene Insensitive 3) and downstream gene expression in alleviating germination inhibition and growth damage induced by high salt. The *Arabidopsis* lines affected in ethylene signaling (*ein3eil1*) and NO biosynthesis (*nia1nia2*) were employed to compare with the wild-type Col-0 and overexpressing line *EIN3ox*. Firstly, the obviously inhibited germination, greater ratio of bleached leaves and enhanced electrolyte leakage were found in *ein3eil1* and *nia1nia2* lines than in Col-0 plants upon high salinity. However, the line *EIN3ox* obtained a notably elevated ability to germinate and improved seedling resistance. The experiment with SNP alone or plus high salt mostly enhanced the expression of *EIN3* transcripts, compared with *ACO4* and *ACS2*. The western blot and transcript analysis found that high-salt-induced EIN3 stabilization and *EIN3* transcripts were largely attenuated in the NO biogenesis mutant *nia1nia2* plants than in Col-0 ones. This observation was confirmed by simulation experiments with NO scavenger cPTIO to block NO emission. Taken together, our study provides insights that NO promotes seed germination and seedlings growth under salinity may depend on EIN3 protein.

## Introduction

Nitric oxide (NO) is a small ubiquitous molecule, which is not only involved in the promotion of seed germination and cell death, shaping of root architecture, senescence, flowering, but also involved in responses to abiotic and biotic stresses, such as drought, salt, heat stress, disease resistance and apoptosis (Zhao et al., 2004; Bethke et al., 2006, 2007; Zhang et al., 2006; Hebelstrup et al., 2012; Arc et al., 2013a,b; Sanz et al., 2015). Two main enzyme-based pathways have been proposed to be functional for NO biosynthesis in plants (Lozano-Juste and Leon, 2010). One is based on the activity of nitrate reductase, which primarily catalyses the reduction of nitrate to nitrite and is encoded by two genes in *Arabidopsis*, designated NIA1 and NIA2 (Meyer et al., 2005; Modolo et al., 2005). Another is based on AtNOS1, although AtNOS1 was originally identified as a NOS-like enzyme that produces NO from L-arginine (Guo and Crawford, 2005), further studies confirmed that AtNOS1 lacked its originally reported NOS activity (Zemojtel et al., 2006).

The gaseous phytohormone ethylene serves as a diffusible hormone in plants (Bleecker and Kende, 2000). Ethylene responses are mediated through a signal transduction cascade in *Arabidopsis thaliana*, including five receptors to the single negative regulator CTR1 and two downstream positive components, EIN2 and EIN3 (Ethylene Insensitive 3) (Chao et al., 1997; Johnson and Ecker, 1998; Alonso et al., 1999; Chang and Stadler, 2001; Guo and Ecker, 2003). EIN3 is a plant-specific nuclear transcription factor that initiates downstream transcriptional cascades for ethylene responses (Yoo et al., 2008). EIN3 was reported to be both necessary and sufficient for the activation of the ethylene pathway. ERF1 (Ethylene Response Factors) is the target of EIN3, which is sufficient and indispensable for ERF1 expression (Solano et al., 1998). Ethylene has long been regarded as a stress hormone. It not only regulates developmental processes such as inhibition of cell expansion, induction of fruit ripening, petal and leaf abscission, organ senescence, but also adapt to stress conditions including salt stress and pathogen responses (Bleecker and Kende, 2000; Chang and Stadler, 2001; Achard et al., 2006).

Salinity is one of the major abiotic stresses that adversely affect crop productivity and quality (Munns, 2002). It was reported that salt stress not only delayed the germination process, reduced the germination rate, but also inhibited the formation of plant seedling after germination (Kim and Park, 2008; Lee et al., 2010). Plants have a complex and sophisticated network to antagonize the seed germination inhibition caused by salt stress and many plant hormones and signaling molecules are involved in the process (Achard et al., 2006; Tanou et al., 2009; Mur et al., 2013). The previous researchers proved that the small signaling molecules ABA, GA, SA, NO, and ETH were all directly involved in the regulation of seed germination inhibition or promotion under salt stress (Gniazdowska et al., 2007; Kim and Park, 2008; Lee et al., 2010; Arc et al., 2013a,b; Jiang et al., 2013; Lin et al., 2013a). Ethylene was proposed to release the impaired seed germination and seedling growth through crosstalk with ABA, ROS, and NO during plant response to stress conditions (Linkies et al., 2009; Asensi-Fabado et al., 2012; Lin et al., 2012; Arc et al., 2013b). NO could decrease ABA and GA ratio in seeds necessary to release inhibition of seed germination induced by salinity or break the dormancy (Bethke et al., 2007). Recent report showed that NO regulates hydrogen peroxide homeostasis in plants through interplaying with ascorbate peroxidase activity during plant stress responses (Lindermayr and Durner, 2015; Yang et al., 2015).

The interaction between ethylene biogenesis and NO has been increasingly attractive (Lin et al., 2013a; Lockhart, 2013; Sanz et al., 2015; Tanou et al., 2015). It has been reported that NO signaling in seeds could principally rely on PTM of specific proteins such as ACS and ACO by S-nitrosylation (Hebelstrup et al., 2012). On the other hand, ethylene biosynthesis can be reversibly inhibited by NO through S-nitrosylation of methionine adenosyltransferase, leading to the reduction of the S-AdoMet pool (Lindermayr et al., 2006; Mur et al., 2013). These observations indicated that there was janus face of NO action on ethylene, inductive or suppresive (Mur et al., 2013). We previously reported that application of exogenous ACC (a precursor of ethylene biosynthesis) or sodium nitroprusside (SNP, an NO donor) largely overcame the inhibition of germination induced by salinity (Lin et al., 2013a). Exogenous nitric oxide was reported to improve seed germination in wheat against mitochondrial oxidative damage induced by high salinity (Zheng et al., 2009). Salt stress causes in-planta accumulation of reactive oxygen species (ROS), which can result in oxidative stress and cellular damage (Lockhart, 2013). These reports highlighted a common property that either NO or ethylene could release the inhibited germination induced by salt-produced ROS stress. In addition, EIN3 had been reported to be sufficient to enhance *Arabidopsis* salt tolerance by decreasing ROS accumulation (Peng et al., 2014). The enhanced oxidative stress in the ethylene-insensitive (*ein3-1*) mutant of *Arabidopsis thaliana* exposed to salt stress supported the predominant role of EIN3 (Asensi-Fabado et al., 2012) in the control of ethylene-mediated plant salt responses. In this study, we hypothesize and provide evidence from both genetic and molecular views that NO modulates seed germination and seedling growth under high salinity through regulating EIN3 protein as well as downstream gene responses.

## Results

### Comparative Analysis of Seed Germination Rate Under Salt Stress Among Four Lines of *Arabidopsis*

Salinity delayed seed germination of all four lines (Col-0, *nia1nia2*, *ein3eil1*, and *EIN3ox*) 3 days after treatment. High salinity (150 and 200 mM NaCl) significantly inhibited seed germination rate of *ein3eil1* and *nia1nia2*, however, *EIN3ox* seeds showed obviously higher germination rate after 3 days of salt stress. Under MS and mild salinity, all four lines normally germinated, as the concentration of salt increased, seed germination rate obviously declined. Compared with Col-0 and *EIN3ox*, nearly 35% of *nia1nia2* and *ein3eil1* seeds germinated on the medium containing 100 mM NaCl, suggesting a significant delay in germination for the *nia1nia2* and *ein3eil1* mutant.

**FIGURE 1:**
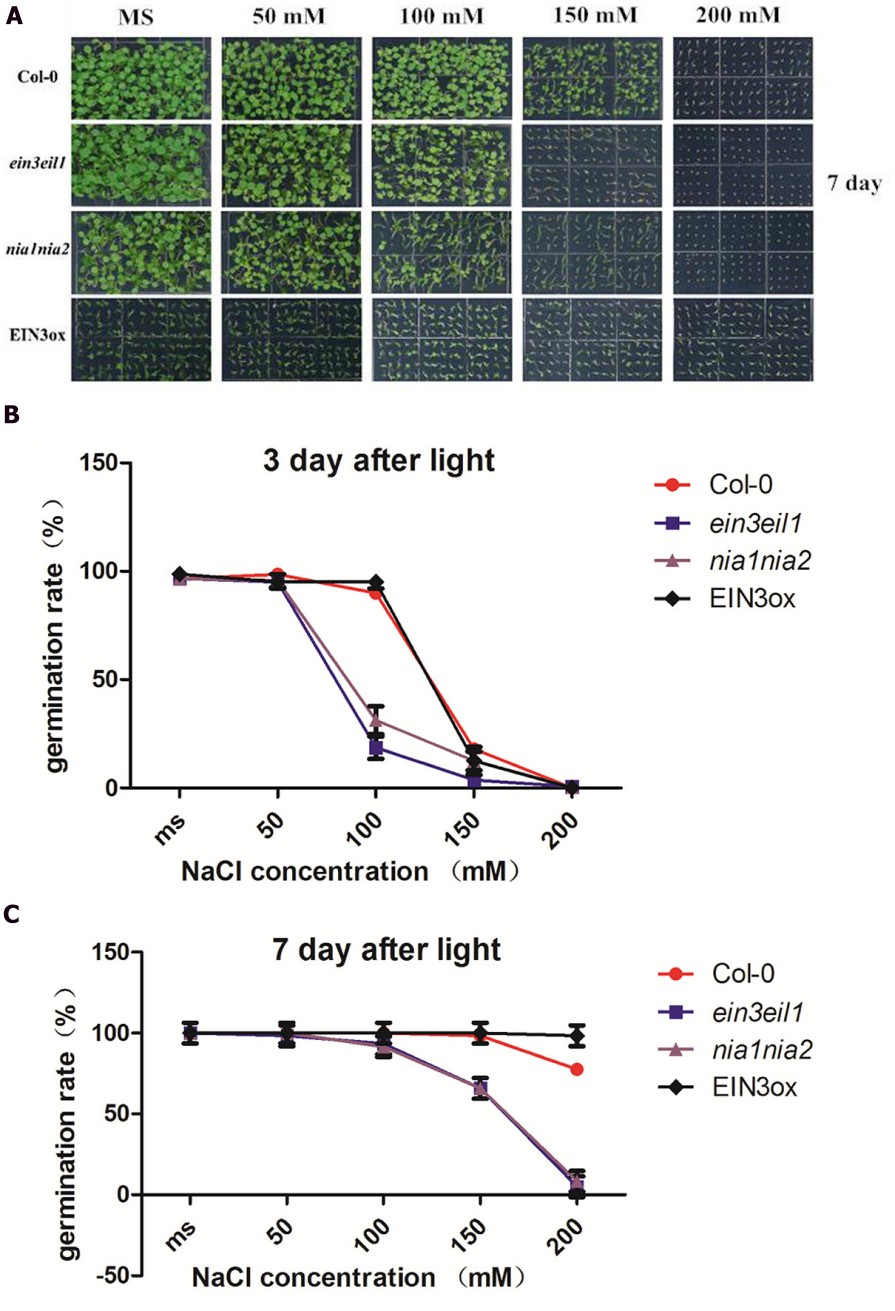
**Comparative analysis of seed germination rate under salt stress among four lines of *Arabidopsis*.** **(A)** Germinating status of Columbia (Col-0), *ein3-1eil1-1, nia1-1nia2-5*, and *EIN3ox* seeds from 0 to 200 mM NaCl concentration plate 7 days after transfer to light. **(B)** Germination rate of Col-0, *ein3-1eil1-1, nia1-1nia2-5*, and *EIN3ox* seeds on 0, 50, 100 150 200 mM NaCl conditions 3 days after transfer to light. **(C)** Germination rate of Col-0, *ein3-1eil1-1, nia1-1nia2-5*, and *EIN3ox* seeds on 0, 50, 100 150 200 mM NaCl conditions 7 days after transfer to light. Data are mean values (SE) of three independent experiments.

When grown on MS and mild salinity (50 and 100 mM NaCl) 7 days after light, no differences in germination rate were observed in the wild-type, *nia1nia2*, *ein3eil1*, and *EIN3ox*. Under high salt stressed conditions (150 and 200 mM NaCl), large differences in germination rate occurred between Col-0 and mutants (*ein3eil1* and *nia1nia2*). High salt stress significantly inhibited *ein3eil1* and *nia1nia2* seed germination, however, nearly all seeds of *EIN3ox* germinated on 200 mM NaCl after 7 days (**Figures 1A,C**).

### Phenotypic and Physiological Analysis of Seedling Tolerance Upon High Salt Alone or Plus NO Scavenger cPTIO

**FIGURE 2:**
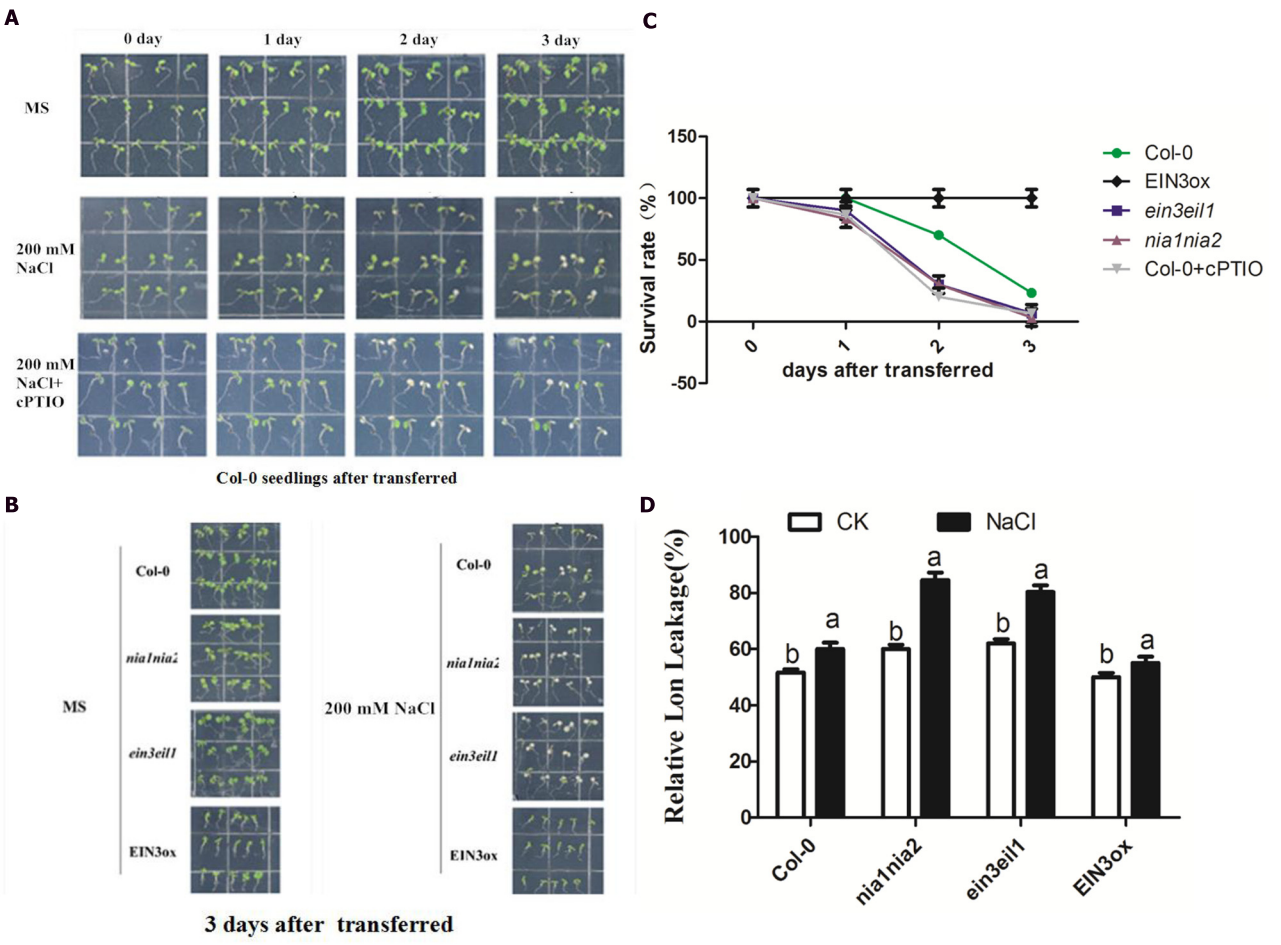
**Phenotypic and physiological analysis of seedling tolerance upon high salt alone or plus NO scavenger cPTIO.** **(A)** High salt generate visible bleached damage in wild type. 5-day-old Col-0 seedlings were continuously treated with 0 mM, 200 mM NaCl and 200 mM NaCl plus 200 μM cPTIO for 3 days. **(B)** High salt alone generates visible bleached damage in Col-0, *ein3-1eil1-1, nia1-1nia2-5*, and *EIN3ox* lines. 5-day-old seedlings were continuously treated with 0 mM NaCl or 200 mM NaCl 3 days. **(C)** The survival rate evaluated by visible bleaching rate. 5-day-old seedlings were treated with 200 mM NaCl for 3 days. **(D)** The comparison of relative electrolyte leakage (REL). 5-day-old seedlings were treated with 200 mM NaCl for 3 days. Data are mean values (SE) of three independent experiments. Within each set of experiments, bars with different letters were significantly different at the 0.05 level (Duncan’s test).

For phenotypic and physiological analysis under salt stress, the 5-day-old seedlings of Col-0, *ein3-1eil1-1*, *nia1-1nia2-5*, and *EIN3ox* were transferred onto MS agar plates containing 200 mM NaCl and their subsequent appearance was recorded photographically 3 days after transfer (Peng et al., 2014). The salt stress damages of seedlings were indicated by visibly bleached leaves and relative electrolyte leakage (REL). The phenotypic observation of Col-0 seedlings after transferred to salinity alone or plus NO scavenger cPTIO showed that the ratio of bleached seedlings obviously increased when cPTIO was supplemented (**Figure 2A**). We then compared the bleached response of four lines of seedlings 3 days after transferred and found that *nia1nia2* and *ein3eil1* mutant showed largely higher ratio of bleached leaves than Col-0 or *EIN3ox* seedlings (**Figure 2B**). The quantitative analysis according to the ratio of bleached to green leaves confirmed the above observations (**Figure 2C**). The REL of leaves under control or salt conditions was further measured to support phenotypic observation. The results showed that salt stress resulted in a higher enhancement of REL in the two mutant seedlings than in the wild-type and *EIN3ox* plants.

### The Salt Induced EIN3 Protein and Transcript Levels Were Modulated by NO Release

**FIGURE 3:**
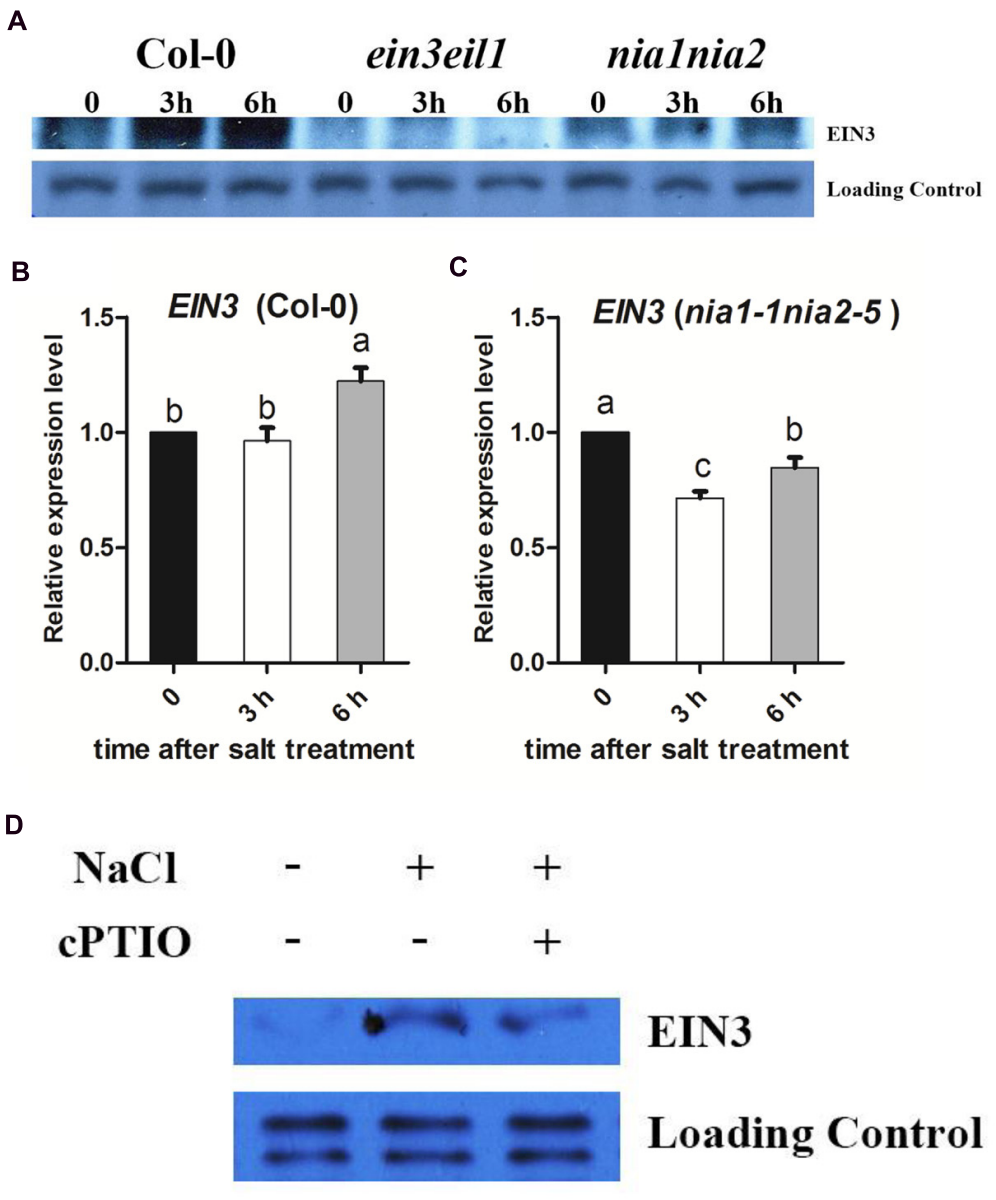
**The salt induced EIN3 protein and transcript levels were modulated by NO release.** **(A)** High salt promotes EIN3 protein accumulation. 5-day-old seedlings of Col-0, *ein3-1eil1-1*, and *nia1-1nia2-5* were treated with 200 mM NaCl for 3 and 6 h. Protein was extracted and subjected to immunoblots using anti-EIN3 antibody. The non-specific band was used as a loading control. **(B)** *EIN3* transcript analysis in wild type. 5-day-old seedlings were treated with 200 mM NaCl for 3 and 6 h. **(C)** *EIN3* transcript analysis in *nia1-1nia2-5*. 5-day-old seedlings were treated with 200 mM NaCl for 3 and 6 h. **(D)** cPTIO impairs high salt induced EIN3 accumulation in wild type Col-0. 5-day-old seedlings were treated with 200 mM NaCl alone or plus 200 μM cPTIO for 6 h. The non-specific band was used as a loading control. Data are mean values (SE) of three independent experiments. Within each set of experiments, bars with different letters were significantly different at the 0.05 level (Duncan’s test).

The ethylene signaling pathway transcription factor EIN3 is a critical regulator of plant salt responses. We found that the *nia1nia2* and *ein3eil1* seeds showed the similar germination rate and survival rate when transferred to MS medium supplemented with salinity (**Figures 1** and **2**). Consequently, EIN3 protein was detected in wild type and both *nia1nia2* and *ein3eil1* mutant. Western blotting results showed that EIN3 protein was dramatically increased after 3 h and 6 h of high salt treatment in wild-type Col-0, and EIN3 protein could not be detected in *ein3eil1* mutant. At the same time, EIN3 protein level in *nia1nia2* plants was slightly increased after 3 h of high salinity, but the abundance accumulated were still far less than Col-0 (**Figure 3A**). *EIN3* mRNA level was also checked and qRT-PCR results showed that there was no obvious change after 3 h of salt treatment and only mild increase after 6 h of salt treatment in Col-0 (**Figure 3B**). The *EIN3* mRNA in *nia1nia2* even decreased after 3 and 6 h of salt treatment (**Figure 3C**).

On this basis, EIN3 protein was also detected to check whether NO scavenger cPTIO affected EIN3 protein accumulation or not under salt stress. Western blotting results showed that EIN3 protein in Col-0 dramatically increased after 6 h of high salt treatment, however, the EIN3 protein level was much lower when treated with high salt plus 200 μM cPTIO (**Figure 3D**).

### The Salt Induced *ERF1* Transcripts were Modulated by NO Release

**FIGURE 4:**
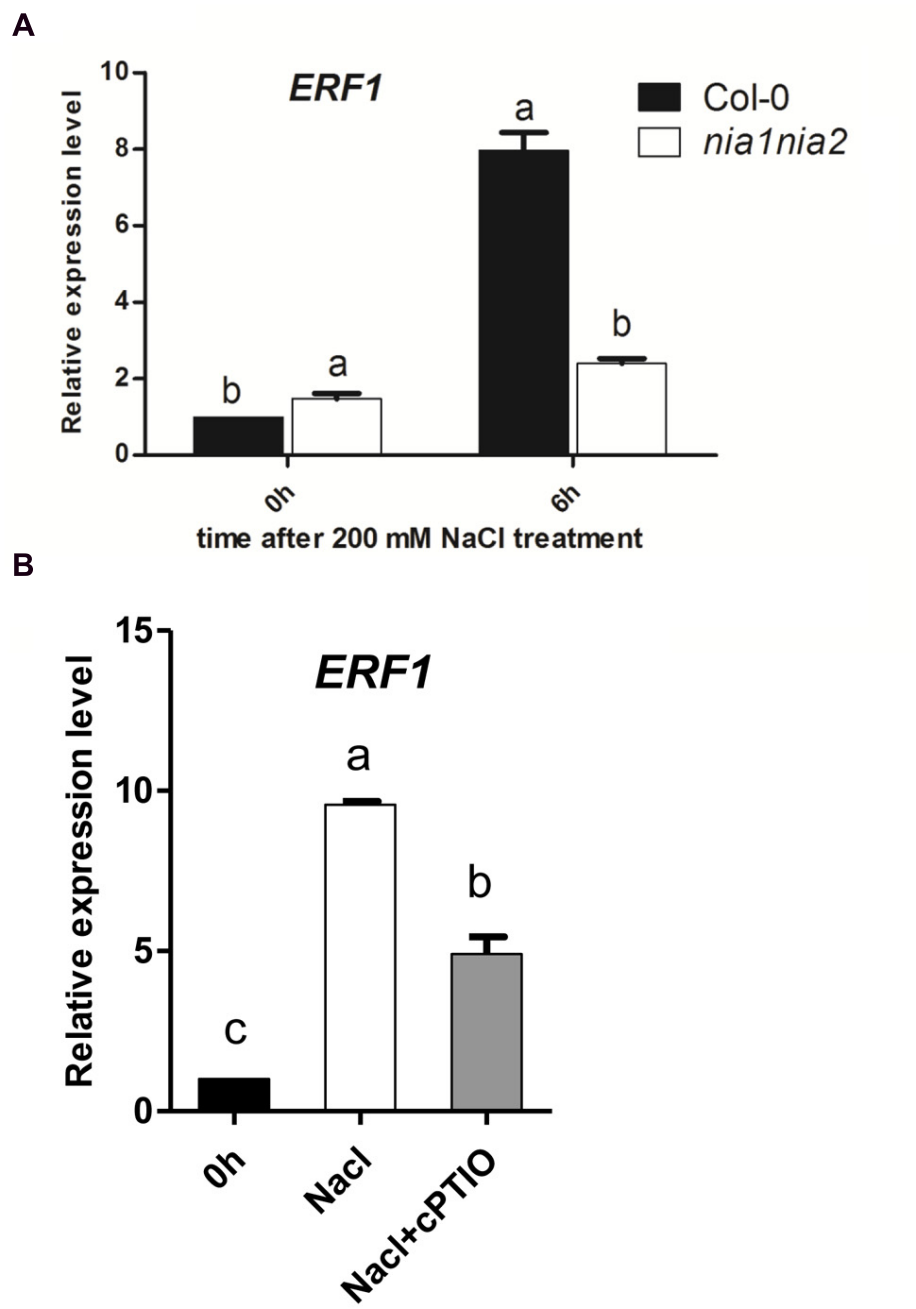
**The salt induced *ERF1* transcripts were modulated by NO release.** **(A)** *ERF1* transcript in Col-0 and *nia1-1nia2-5*. 5-day-old seedlings were treated with 200 mM NaCl for 6 h. **(B)** *ERF1* transcript in Col-0 affected by salt and cPTIO. 5-day-old seedlings were treated with 200 mM NaCl alone or plus cPTIO for 6 h. Data are mean values (SE) of three independent experiments. Within each set of experiments, bars with different letters were significantly different at the 0.05 level (Duncan’s test).

To check whether the accumulation EIN3 protein is due to transcriptional function or not, the expression levels of the representative ethylene responsive genes *ERF1* were measured. ERF1 transcript was up-regulated by high salt both in wild type Col-0 and *nia1nia2* mutant, however, the increased amount of *ERF1* expression in *nia1nia2* is much lower than that in Col-0. The expression level of *ERF1* increased eightfold after 6 h of high salt treatment, which coincided with the EIN3 protein level, however, in *nia1nia2* mutants, 6 h high salt treatment only increased *ERF1* expression about twofold (**Figure 4A**).

Nitric oxide scavenger cPTIO were also used to treat wild type Col-0 and then check the *ERF1* expression in seedlings affected by NO release under salt stress. The results showed that *ERF1* gene expression was up-regulated by 200 mM NaCl treatment in wild type plants, which was consistent with the above-mentioned results (**Figure 4A**). The expression level of *ERF1* increased approximately ninefold after 6 h of high salt treatment, however, the expression level of *ERF1* decreased when seedlings were treated with high salt plus 200 μM cPTIO compared with high salt alone. In this case, the expression level of *ERF1* only increased fourfold, which coincided with the EIN3 protein level observed (**Figure 4B**).

### Transcriptional Comparisons of *ACO4*, *ACS2*, and *EIN3* Affected by High Salt and NO Donor

**FIGURE 5:**
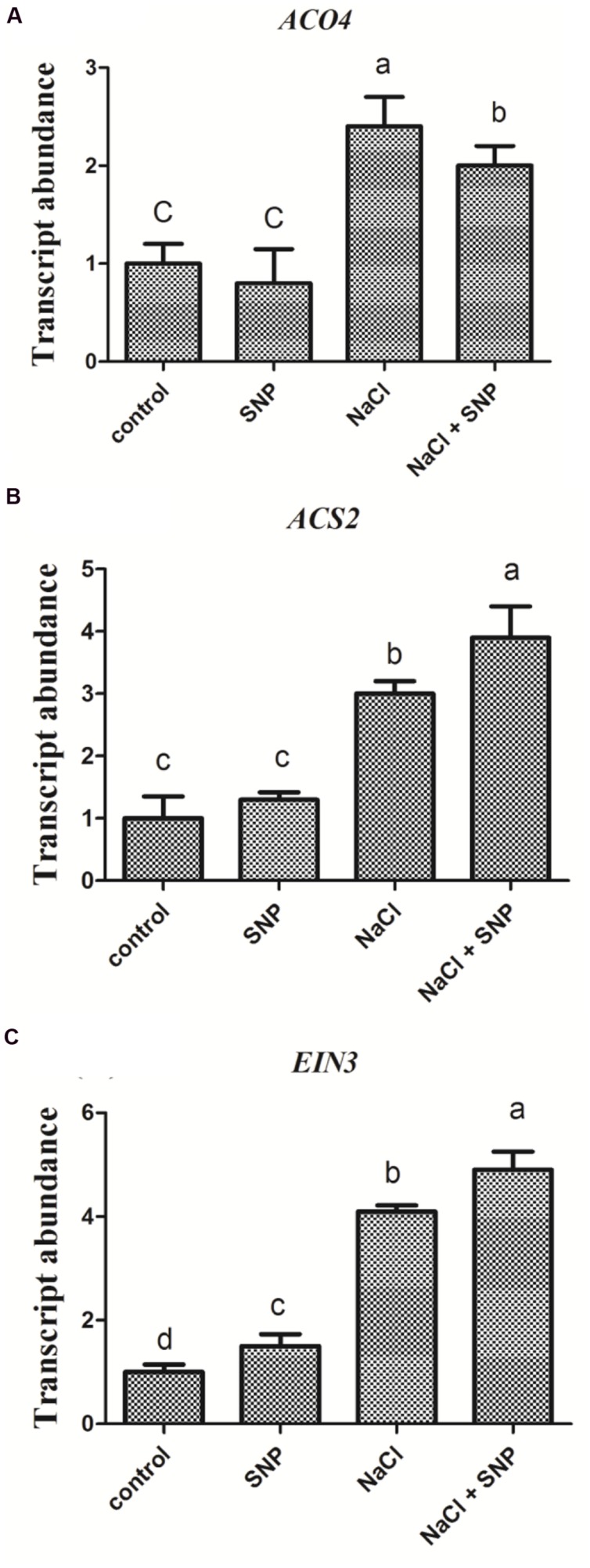
**Transcriptional comparison of *ACO4*, *ACS2*, and *EIN3* in Col-0 seedlings affected by high salt and NO donor.** 5-day-old seedlings were exposed to four treatments 0 mM NaCl, 50 μM SNP, 200 mM NaCl, and 200 mM NaCl plus 50 μM SNP for 6 h. Then the seedlings were subjected to qRT-PCR analysis to compare the transcriptional abundance of *ACO4* **(A)***, ACS2* **(B)**, and *EIN3* **(C)**. Data are mean values (SE) of three independent experiments. Within each set of experiments, bars with different letters were significantly different at the 0.05 level (Duncan’s test).

Compared to the control, no significant changes were observed in transcript levels of ethylene-synthesis related genes (*ACO4* and *ACS2*) in Col-0 seedlings treated with SNP (50 μM) alone. However, the expression of *ACO4* and *ACS2* genes were increased markedly following salt stress. Furthermore, when SNP (50 μM) was applied to NaCl stressed seedlings at the same time, the expression of *ACO4* and *ACS2* genes were greatly induced compared to that under salinity alone (**Figures 5A,B**). On the contrary, treatment with SNP alone specifically enhanced the expression of *EIN3* gene compared to *ACO4* and *ACS2*. Treatment with salt and SNP together further increased *EIN3* gene expression compared with the control (**Figure 5C**).

## Discussion

A critical role of NO in dormancy break or germination promotion has been demonstrated in many plants under optimal or stressed conditions (Bewley, 1997; Bethke et al., 2006, 2007; Libourel et al., 2006; Liu et al., 2009; Lin et al., 2013a; Lindermayr and Durner, 2015). NO is considered as a likely player of a signaling pathway that promotes loss of dormancy and has been suggested to behave as an endogenous regulator of this process (Arc et al., 2013a). The involved mechanisms had been widely investigated. A great deal of work proposed that NO-induced rapid decrease of abscisic acid concentration was required in breaking seed dormancy in *Arabidopsis* (Liu et al., 2009; Arc et al., 2013b; Wang et al., 2015). NO was demonstrated to act with ABA 8′-hydroxylase, resulting in ABA catabolism and breaking seed dormancy in *Arabidopsis* (Liu et al., 2009; Arc et al., 2013b). Increasing work indicated that NO could also interact with ethylene biosynthesis or signaling to break seed dormancy or improve salt tolerance. Dormancy removal in apple embryos by nitric oxide or cyanide involves modifications in ethylene biosynthetic pathway (Gniazdowska et al., 2007, 2010). In addition, the up-accumulation of NO under hypoxia can stimulate ethylene biosynthesis, possibly through PTM of key enzymes such as ACS and ACO by *S*-nitrosylation (Hebelstrup et al., 2012). Our previous study showed that NO and ethylene cooperated in enhancing seed germination of *Arabidopsis* under salinity (Lin et al., 2013a). All these evidence suggested a close crosstalk between NO and ethylene during plant growth and stress response. However, the directly functioning targets of NO in ethylene signaling pathway remain unclear.

Ethylene is a major phytohormone that regulates plant development in response to adverse environments (Wang et al., 2002; Guo and Ecker, 2003; Potuschak et al., 2003; Zhu et al., 2011). In ethylene signaling transduction pathway, EIN3 protein is the key transcription factor and EIN3/EIL1 (EIN3-LIKE 1) are both necessary and sufficient in the activation of transcription of ERF1 and other downstream genes (Guo and Ecker, 2003). The *ein3-1eil1-1* double mutant exhibited remarkably reduced tolerance to high concentration of salt (Peng et al., 2014) and so this mutant was employed in this report. The experiment with EIN3-overexpressing (*EIN3ox*) line plants revealed that EIN3 was sufficient to enhance transcript levels of salt-related genes and salt tolerance (Zhang et al., 2011). Another important component in this pathway is EIN2 protein, which is in the upstream of EIN3 and positively regulates the functions of EIN3/EIL1. We previously reported that loss-of-function mutation of EIN2 protein in *Arabidopsis* exaggerated oxidative stress induced by salinity (Lin et al., 2013b). However, EIN2 was recently reported to be not necessary during high salinity, since that high salinity enhances EIN3 protein accumulation and transcriptional activity in both EIN2-dependent and EIN2-independent manners (Peng et al., 2014). NO was proposed to participate in diverse biological process through S-nitrosylation of nuclear proteins (Kovacs and Lindermayr, 2013; Mengel et al., 2013). It is coincident to find that EIN3/EIL1 are also stabilized and accumulated in the nucleus (Chao et al., 1997). These findings raise the assumption in this report that EIN3 is another NO targeted nuclear transcriptional factor by *S*-nitrosylation during their interplaying to release salt-induced seed germination inhibition.

Based on the above-mentioned four lines of *Arabidopsis*, we firstly compared the germination rate and seedling tolerance of *ein3-1eil1-1, nia1-1nia2-5*, and *EIN3ox* plants with the wild-type under salt conditions. Comparatively, the obviously decreased germination rate, greater ratio of bleached leaves and enhanced electrolyte leakage were found in *ein3eil1* and *nia1nia2* lines than in Col-0 plants upon high salinity. However, the line *EIN3ox* obtained a notably elevated ability to totally germinate and improved seedling resistance under high salt conditions. We then treated wild type Col-0 with NO scavenger cPTIO to mimic *nia1nia2* mutant and the consistent results were achieved. It was reported that NO donor SNP greatly induced the expression of the ACS2 gene (Garcia et al., 2010; Lin et al., 2013a). Our experiment with SNP alone or plus high salt mostly enhanced the expression of *EIN3* transcripts, compared with *ACO4* and *ACS2*. These observations and reports then displayed a close crosstalk of NO with EIN3 protein to improve high salt resistance in *Arabidopsis*.

EIN3 protein level and downstream gene expression were then checked in the salt-stressed seedlings. We found that the high salt stabilized EIN3 protein accumulation and *EIN3* transcripts were largely attenuated in the NO biogenesis mutant *nia1nia2* plants than in Col-0 ones. For *ein3eil1* line, EIN3 protein could not be detected under control or salt condition. Our data showed NO biogenesis mutant *nia1nia2* impair EIN3 protein accumulation and transcript, especially induced by high salt. Our simulation experiments with NO scavenger cPTIO to block NO emission confirmed the above results with mutant plants. This evidence verifies that loss of NO will inhibit EIN3 level and gene transcripts under salt stress. The function of ethylene is finally accomplished by the signaling transduction especially the key transcription factor EIN3 protein level and downstream gene expression (Rieu et al., 2003; Asensi-Fabado et al., 2012). The classic EIN3 downstream ethylene responsive gene *ERF1* transcript was simultaneously detected and the changes coincided with EIN3 protein and its transcript in salt-stressed Col-0 and *nia1nia2* plants. All above data clearly indicates that NO modulates the ethylene signaling pathway transcription factor EIN3 accumulation to counteract salt stress.

Nitric oxide had been increasingly reported to modulate numerous cellular functions in plants through protein post-translational modifications (PTMs) of nuclear enzyme activities (Lindermayr et al., 2006; Tanou et al., 2009; Kovacs and Lindermayr, 2013; Mengel et al., 2013; Wang et al., 2015). The ethylene biosynthesis enzyme ACS (Lindermayr et al., 2006), ascorbate peroxidase (Yang et al., 2015) and OST1 (open stomata 1) (Wang et al., 2015) had all been successfully identified to be the *S*-nitrosylated substrates. In this way, NO establishes a comprehensive crosstalk network with other signaling molecules, such as ABA, JA, ethylene, ROS to regulate plant growth and defense (Abat and Deswal, 2009; Tanou et al., 2009; Manjunatha et al., 2012; Kovacs and Lindermayr, 2013; Wang et al., 2015). Here, we propose that NO promotes seed germination and seedling growth under high salt condition may depend on EIN3 protein accumulation and the downstream targeted responsive gene in *Arabidopsis*. Further study need to investigate whether NO also modify EIN3 activity through *S*-nitrosylation, which had been found in the way of NO acting on ACS, ACO activities in plants.

## Materials and Methods

### Plant Materials and Growth Conditions

The wild type was Columbia ecotype (Col-0), an *ein3* and *eil1* double mutant (*ein3-1eil1-1*) (Alonso et al., 1999), and a transgenic line overexpressing EIN3 (*EIN3ox*) (Chao et al., 1997) and SALK line *nia1nia2* (*nia1-1nia2-5*) were used in the study. Surface-sterilized seeds were sown on Murashige and Skoog (MS) medium (4.3 g/L MS salts, 10 g/L Suc, pH 5.7 to 5.8, and 8 g/L agar) imbibed at 4°C for 3 days in the dark and then germinated at 23°C under a 16/8 light/dark regime.

### Assay of Seed Germination Rate

For germination assays, at least 120 seeds per plate from Col-0, *ein3-1eil1-1*, *nia1nia2*, and *EIN3ox* plants and three replicates of each were sowed onto MS medium supplemented with various concentrations of NaCl (50, 100, 150, and 200 mM). Germination was assessed from 3rd days to 7th days after transfer to light. A seed was considered as germinated when the radical protruded through the envelopes.

### Seedling Survival Rate and Relative Electrolyte Leakage Assays

For bleaching analysis of salt-stressed leaves, 5 days seedlings were transferred onto MS agar plates containing 200 mM NaCl alone or plus 200 μM cPTIO and their subsequent appearance was recorded photographically 3 days after transferred. The seedling survival rate was simultaneously scored as bleaching ratio of leaves (Peng et al., 2014). Leaf REL were measured as described previously (Peever and Higgins, 1989).

### Protein Extracting and Western Blotting Assays

Five days seedlings of Col-0, *ein3-1eil1-1* and *nia1-1nia2-5* were treated with 200 mM NaCl for 3 and 6 h. Protein was extracted and subjected to immunoblots using anti-EIN3 antibody. Plant samples were ground in liquid N2 and soluble protein extracts were made by homogenization in 50 mM Tris–HCl (pH 8.0), 10 mM NaCl, 0.1 M PMSF, and 0.1 M DTT, with subsequent centrifugation at 13000 *g* for 30 min at 4°C The protein in the supernatants was quantified by Bradford’s assay (Bradford, 1976). Western blot analysis was performed as described previously (Alonso et al., 1999) with anti-EIN3 antibodies (Guo and Ecker, 2003).

### qRT-PCR Analysis of Gene Expression

For expression profiling of ethylene-related genes in seedlings under salt stress in the presence of SNP or cPTIO was used as the NO donor and inhibitor. The seedlings were transferred onto MS agar plates containing 200 mM NaCl or 200 mM NaCl plus 50 μM SNP for 3–6 h. There were three replicates of each type of seed treatment plate. The qRT-PCR assay has been designed according to the Minimum Information for Publication of Quantitative Real-Time PCR Experiments (MIQE) guidelines (Remans et al., 2014).

Total RNA was prepared using the TRIzol reagent (Invitrogen). Two micrograms of total RNA treated with DNase I (Promega) was added in a 20 μL reverse transcription reaction using the M-MLV reverse transcription system (Promega). Real-time PCR was performed using SYBR Green Mix (Takara) and the specific primers:

UBQ10-F: TCTCGTCTCTGTTATGCTTAAGAAG,
UBQ10-R: AGAAAGAAAGAGATAACAGGAACGG,
ERF1-F: ACCGCTCCGTGAAGTTAGATAATG,
ERF1-R: ATCCTAATCTTTCACCAAGTCCCAC,
EIN3-F: TGGAGAGACAAAATGCGGCT,
EIN3-R: ATAGCCGCAGGACCATTACG,
ACS2-F: AGGCAATTGCACATTTCATGG,
ACS2-R: CTGTCCGCCACCTCAAGTCT,
ACO4-F: CCGATGTCCCTGATCTCGAC,
ACO4- R: AGTCGGTCTTTTCGACCCGTA,

The expression level was normalized to that of the UBIQUITIN10 (UBQ10) control. The primer efficiency was calculated from qRT-PCR of the serial dilution of total cDNA, and the specificity of the primers was confirmed by the dissociation curve for each primer set in accordance with the MIQE guidelines.

### Statistical Analysis

Statistical treatment was carried out by analysis of variance (ANOVA) using SPSS-19 statistical software package. Results were represented as the means ± SE. Differences between treatments were separated by Duncan multiple range test at a 0.05 probability level.

## Conflict of Interest Statement

The authors declare that the research was conducted in the absence of any commercial or financial relationships that could be construed as a potential conflict of interest.

## References

[B1] Abat J. K. Deswal R. (2009). Differential modulation of S-nitrosoproteome of *Brassica juncea* by low temperature: change in S-nitrosylation of Rubisco is responsible for the inactivation of its carboxylase activity. *Proteomics* 9 4368–4380. 10.1002/pmic.200800985 19655309

[B2] Achard P. Cheng H. De Grauwe L. Decat J. Schoutteten H. Moritz T. (2006). Integration of plant responses to environmentally activated phytohormonal signals. *Science* 311 91–94. 10.1126/science.1118642 16400150

[B3] Alonso J. M. Hirayama T. Roman G. Nourizadeh S. Ecker J. R. (1999). EIN2, a bifunctional transducer of ethylene and stress responses in *Arabidopsis*. *Science* 284 2148–2152. 10.1126/science.284.5423.2148 10381874

[B4] Arc E. Galland M. Godin B. Cueff G. Rajjou L. (2013a). Nitric oxide implication in the control of seed dormancy and germination. *Front. Plant Sci.* 4:346 10.3389/fpls.2013.00346 PMC3777103 24065970

[B5] Arc E. Sechet J. Corbineau F. Rajjou L. Marion-Poll A. (2013b). ABA crosstalk with ethylene and nitric oxide in seed dormancy and germination. *Front. Plant Sci.* 4:63 10.3389/fpls.2013.00063 PMC3607800 23531630

[B6] Asensi-Fabado M. A. Cela J. Muller M. Arrom L. Chang C. Munne-Bosch S. (2012). Enhanced oxidative stress in the ethylene-insensitive (ein3-1) mutant of *Arabidopsis thaliana* exposed to salt stress. *J. Plant Physiol.* 169 360–368. 10.1016/j.jplph.2011.11.007 22209220

[B7] Bethke P. C. Libourel I. G. Aoyama N. Chung Y. Y. Still D. W. Jones R. L. (2007). The *Arabidopsis aleurone* layer responds to nitric oxide, gibberellin, and abscisic acid and is sufficient and necessary for seed dormancy. *Plant Physiol.* 143 1173–1188. 10.1104/pp.106.093435 17220360 PMC1820924

[B8] Bethke P. C. Libourel I. G. Jones R. L. (2006). Nitric oxide reduces seed dormancy in *Arabidopsis*. *J. Exp. Bot.* 57 517–526. 10.1093/jxb/erj060 16377732

[B9] Bewley J. D. (1997). Seed germination and dormancy. *Plant Cell* 9 1055–1066. 10.1105/tpc.9.7.1055 12237375 PMC156979

[B10] Bleecker A. B. Kende H. (2000). Ethylene: a gaseous signal molecule in plants. *Annu. Rev. Cell Dev. Biol.* 16 1–18. 10.1146/annurev.cellbio.16.1.1 11031228

[B11] Bradford M. M. (1976). A rapid and sensitive method for the quantitation of microgram quantities of protein utilizing the principle of protein-dye binding. *Anal. Biochem.* 72 248–254. 10.1016/0003-2697(76)90527-3 942051

[B12] Chang C. Stadler R. (2001). Ethylene hormone receptor action in *Arabidopsis*. *Bioessays* 23 619–627. 10.1002/bies.1087 11462215

[B13] Chao Q. Rothenberg M. Solano R. Roman G. Terzaghi W. Ecker J. R. (1997). Activation of the ethylene gas response pathway in *Arabidopsis* by the nuclear protein ETHYLENE-INSENSITIVE3 and related proteins. *Cell* 89 1133–1144. 10.1016/S0092-8674(00)80300-1 9215635

[B14] Garcia M. J. Lucena C. Romera F. J. Alcantara E. Perez-Vicente R. (2010). Ethylene and nitric oxide involvement in the up-regulation of key genes related to iron acquisition and homeostasis in *Arabidopsis*. *J. Exp. Bot.* 61 3885–3899. 10.1093/jxb/erq203 20627899

[B15] Gniazdowska A. Dobrzynska U. Babanczyk T. Bogatek R. (2007). Breaking the apple embryo dormancy by nitric oxide involves the stimulation of ethylene production. *Planta* 225 1051–1057. 10.1007/s00425-006-0384-z 17260145

[B16] Gniazdowska A. Krasuska U. Bogatek R. (2010). Dormancy removal in apple embryos by nitric oxide or cyanide involves modifications in ethylene biosynthetic pathway. *Planta* 232 1397–1407. 10.1007/s00425-010-1262-2 20830596

[B17] Guo F. Q. Crawford N. M. (2005). *Arabidopsis* nitric oxide synthase1 is targeted to mitochondria and protects against oxidative damage and dark-induced senescence. *Plant Cell* 17 3436–3450. 10.1105/tpc.105.037770 16272429 PMC1315380

[B18] Guo H. Ecker J. R. (2003). Plant responses to ethylene gas are mediated by SCF(EBF1/EBF2)-dependent proteolysis of EIN3 transcription factor. *Cell* 115 667–677. 10.1016/S0092-8674(03)00969-3 14675532

[B19] Hebelstrup K. H. van Zanten M. Mandon J. Voesenek L. A. C. J. Harren F. J. M. Cristescu S. M. (2012). Haemoglobin modulates NO emission and hyponasty under hypoxia-related stress in *Arabidopsis thaliana*. *J. Exp. Bot.* 63 5581–5591. 10.1093/jxb/ers210 22915746 PMC3444272

[B20] Jiang C. Belfield E. J. Cao Y. Smith J. A. Harberd N. P. (2013). An *Arabidopsis* soil-salinity-tolerance mutation confers ethylene-mediated enhancement of sodium/potassium homeostasis. *Plant Cell* 25 3535–3552. 10.1105/tpc.113.115659 24064768 PMC3809548

[B21] Johnson P. R. Ecker J. R. (1998). The ethylene gas signal transduction pathway: a molecular perspective. *Annu. Rev. Genet.* 32 227–254. 10.1146/annurev.genet.32.1.227 9928480

[B22] Kim S. G. Park C. M. (2008). Gibberellic acid-mediated salt signaling in seed germination. *Plant Signal. Behav.* 3 877–879. 10.4161/psb.3.10.6247 19704528 PMC2634403

[B23] Kovacs I. Lindermayr C. (2013). Nitric oxide-based protein modification: formation and site-specificity of protein S-nitrosylation. *Front. Plant Sci.* 4:137 10.3389/fpls.2013.00137 PMC3653056 23717319

[B24] Lee S. Kim S. G. Park C. M. (2010). Salicylic acid promotes seed germination under high salinity by modulating antioxidant activity in *Arabidopsis*. *New Phytol.* 188 626–637. 10.1111/j.1469-8137.2010.03378.x 20663063

[B25] Libourel I. G. Bethke P. C. De Michele R. Jones R. L. (2006). Nitric oxide gas stimulates germination of dormant *Arabidopsis* seeds: use of a flow-through apparatus for delivery of nitric oxide. *Planta* 223 813–820. 10.1007/s00425-005-0117-8 16172867

[B26] Lin Y. Yang L. Paul M. Zu Y. Tang Z. (2013a). Ethylene promotes germination of *Arabidopsis* seed under salinity by decreasing reactive oxygen species: evidence for the involvement of nitric oxide simulated by sodium nitroprusside. *Plant Physiol. Biochem.* 73 211–218. 10.1016/j.plaphy.2013.10.003 24148906

[B27] Lin Y. C. Chen D. D. Paul M. Zu Y. G. Tang Z. H. (2013b). Loss-of-function mutation of EIN2 in *Arabidopsis* exaggerates oxidative stress induced by salinity. *Acta Physiol. Plant.* 35 1319–1328. 10.1007/s11738-012-1172-y

[B28] Lin Y. C. Wang J. J. Zu Y. G. Tang Z. H. (2012). Ethylene antagonizes the inhibition of germination in *Arabidopsis* induced by salinity by modulating the concentration of hydrogen peroxide. *Acta Physiol. Plant.* 34 1895–1904. 10.1007/s11738-012-0989-8

[B29] Lindermayr C. Durner J. (2015). Interplay of reactive oxygen species and nitric oxide: nitric oxide coordinates reactive oxygen species homeostasis. *Plant Physiol.* 167 1209–1210. 10.1104/pp.15.00293 25819986 PMC4378185

[B30] Lindermayr C. Saalbach G. Bahnweg G. Durner J. (2006). Differential inhibition of *Arabidopsis* methionine adenosyltransferases by protein S-Nitrosylation. *J. Biol. Chem.* 281 4285–4291. 10.1074/jbc.M511635200 16365035

[B31] Linkies A. Muller K. Morris K. Tureckova V. Wenk M. Cadman C. S. (2009). Ethylene interacts with abscisic acid to regulate endosperm rupture during germination: a comparative approach using *Lepidium sativum* and *Arabidopsis thaliana*. *Plant Cell* 21 3803–3822. 10.1105/tpc.109.070201 20023197 PMC2814513

[B32] Liu Y. Shi L. Ye N. Liu R. Jia W. Zhang J. (2009). Nitric oxide-induced rapid decrease of abscisic acid concentration is required in breaking seed dormancy in *Arabidopsis*. *New Phytol.* 183 1030–1042. 10.1111/j.1469-8137.2009.02899.x 19522839

[B33] Lockhart J. (2013). Salt of the earth: ethylene promotes salt tolerance by enhancing Na/K homeostasis. *Plant Cell* 25:3150 10.1105/tpc.113.250911 PMC3809522 24064767

[B34] Lozano-Juste J. Leon J. (2010). Enhanced abscisic acid-mediated responses in nia1nia2noa1-2 triple mutant impaired in NIA/NR- and AtNOA1-dependent nitric oxide biosynthesis in *Arabidopsis*. *Plant Physiol.* 152 891–903. 10.1104/pp.109.148023 20007448 PMC2815865

[B35] Manjunatha G. Gupta K. J. Lokesh V. Mur L. A. Neelwarne B. (2012). Nitric oxide counters ethylene effects on ripening fruits. *Plant Signal. Behav.* 7 476–483. 10.4161/psb.19523 22499176 PMC3419037

[B36] Mengel A. Chaki M. Shekariesfahlan A. Lindermayr C. (2013). Effect of nitric oxide on gene transcription - S-Nitrosylation of nuclear proteins. *Front. Plant Sci.* 4:293 10.3389/fpls.2013.00293 PMC3729996 23914201

[B37] Meyer C. Lea U. S. Provan F. Kaiser W. M. Lillo C. (2005). Is nitrate reductase a major player in the plant NO (nitric oxide) game? *Photosynth. Res.* 83 181–189. 10.1007/s11120-004-3548-3 16143851

[B38] Modolo L. V. Augusto O. Almeida I. M. Magalhaes J. R. Salgado I. (2005). Nitrite as the major source of nitric oxide production by *Arabidopsis thaliana* in response to *Pseudomonas syringae*. *FEBS Lett.* 579 3814–3820. 10.1016/j.febslet.2005.05.078 15978583

[B39] Munns R. (2002). Comparative physiology of salt and water stress. *Plant Cell Environ.* 25 239–250. 10.1046/j.0016-8025.2001.00808.x 11841667

[B40] Mur L. A. Prats E. Pierre S. Hall M. A. Hebelstrup K. H. (2013). Integrating nitric oxide into salicylic acid and jasmonic acid/ ethylene plant defense pathways. *Front. Plant Sci.* 4:215 10.3389/fpls.2013.00215 PMC3694216 23818890

[B41] Peever T. L. Higgins V. J. (1989). Electrolyte leakage, lipoxygenase, and lipid peroxidation induced in tomato leaf tissue by specific and nonspecific elicitors from *Cladosporium fulvum.* *Plant Physiol.* 90 867–875. 10.1104/pp.90.3.867 16666890 PMC1061813

[B42] Peng J. Li Z. Wen X. Li W. Shi H. Yang L. (2014). Salt-induced stabilization of EIN3/EIL1 confers salinity tolerance by deterring ROS accumulation in *Arabidopsis*. *PLoS Genet.* 10:e1004664 10.1371/journal.pgen.1004664 PMC4199496 25330213

[B43] Potuschak T. Lechner E. Parmentier Y. Yanagisawa S. Grava S. Koncz C. (2003). EIN3-dependent regulation of plant ethylene hormone signaling by two *Arabidopsis* F box proteins: EBF1 and EBF2. *Cell* 115 679–689. 10.1016/S0092-8674(03)00968-1 14675533

[B44] Remans T. Keunen E. Bex G. J. Smeets K. Vangronsveld J. Cuypers A. (2014). Reliable gene expression analysis by reverse transcription-quantitative PCR: reporting and minimizing the uncertainty in data accuracy. *Plant Cell* 26 3829–3837. 10.1105/tpc.114.130641 25361954 PMC4247583

[B45] Rieu I. Mariani C. Weterings K. (2003). Expression analysis of five tobacco EIN3 family members in relation to tissue-specific ethylene responses. *J. Exp. Bot.* 54 2239–2244. 10.1093/jxb/erg240 12909687

[B46] Sanz L. Albertos P. Mateos I. Sánchez-Vicente I. Lechón T. Fernández-Marcos M. (2015). Nitric oxide (NO) and phytohormones crosstalk during early plant development. *J. Exp. Bot.* 66 2857–2868. 10.1093/jxb/erv213 25954048

[B47] Solano R. Stepanova A. Chao Q. Ecker J. R. (1998). Nuclear events in ethylene signaling: a transcriptional cascade mediated by ETHYLENE-INSENSITIVE3 and ETHYLENE-RESPONSE-FACTOR1. *Genes Dev.* 12 3703–3714. 10.1101/gad.12.23.3703 9851977 PMC317251

[B48] Tanou G. Job C. Rajjou L. Arc E. Belghazi M. Diamantidis G. (2009). Proteomics reveals the overlapping roles of hydrogen peroxide and nitric oxide in the acclimation of citrus plants to salinity. *Plant J.* 60 795–804. 10.1111/j.1365-313X.2009.04000.x 19682288

[B49] Tanou G. Minas I. S. Karagiannis E. Tsikou D. Audebert S. Papadopoulou K. K. (2015). The impact of sodium nitroprusside and ozone in kiwifruit ripening physiology: a combined gene and protein expression profiling approach. *Ann. Bot.* 116 649–662. 10.1093/aob/mcv107 26159933 PMC4578001

[B50] Wang K. L. Li H. Ecker J. R. (2002). Ethylene biosynthesis and signaling networks. *Plant Cell* 14(Suppl.), S131–S151. 12045274 10.1105/tpc.001768 PMC151252

[B51] Wang P. Du Y. Hou Y. J. Zhao Y. Hsu C. C. Yuan F. (2015). Nitric oxide negatively regulates abscisic acid signaling in guard cells by S-nitrosylation of OST1. *Proc. Natl. Acad. Sci. U.S.A.* 112 613–618. 10.1073/pnas.1423481112 25550508 PMC4299189

[B52] Yang H. Mu J. Chen L. Feng J. Hu J. Li L. (2015). S-nitrosylation positively regulates ascorbate peroxidase activity during plant stress responses. *Plant Physiol.* 167 1604–1615. 10.1104/pp.114.255216 25667317 PMC4378166

[B53] Yoo S. D. Cho Y. H. Tena G. Xiong Y. Sheen J. (2008). Dual control of nuclear EIN3 by bifurcate MAPK cascades in C2H4 signalling. *Nature* 451 789–795. 10.1038/nature06543 18273012 PMC3488589

[B54] Zemojtel T. Frohlich A. Palmieri M. C. Kolanczyk M. Mikula I. Wyrwicz L. S. (2006). Plant nitric oxide synthase: a never-ending story? *Trends Plant Sci.* 11 524–525. 10.1016/j.tplants.2006.09.008 17030145

[B55] Zhang L. Li Z. Quan R. Li G. Wang R. Huang R. (2011). An AP2 domain-containing gene, ESE1, targeted by the ethylene signaling component EIN3 is important for the salt response in *Arabidopsis*. *Plant Physiol.* 157 854–865. 10.1104/pp.111.179028 21832142 PMC3192559

[B56] Zhang Y. Wang L. Liu Y. Zhang Q. Wei Q. Zhang W. (2006). Nitric oxide enhances salt tolerance in maize seedlings through increasing activities of proton-pump and Na^+^/H^+^ antiport in the tonoplast. *Planta* 224 545–555. 10.1007/s00425-006-0242-z 16501990

[B57] Zhao L. Zhang F. Guo J. Yang Y. Li B. Zhang L. (2004). Nitric oxide functions as a signal in salt resistance in the calluses from two ecotypes of reed. *Plant Physiol.* 134 849–857. 10.1104/pp.103.030023 14739346 PMC344559

[B58] Zheng C. Jiang D. Liu F. Dai T. Liu W. Jing Q. (2009). Exogenous nitric oxide improves seed germination in wheat against mitochondrial oxidative damage induced by high salinity. *Environ. Exp. Bot.* 67 222–227. 10.1016/j.envexpbot.2009.05.002

[B59] Zhu Z. An F. Feng Y. Li P. Xue L. Jiang Z. (2011). Derepression of ethylene-stabilized transcription factors (EIN3/EIL1) mediates jasmonate and ethylene signaling synergy in *Arabidopsis*. *Proc. Natl. Acad. Sci. U.S.A.* 108 12539–12544. 10.1073/pnas.1103959108 21737749 PMC3145709

